# A semi supervised framework for human and machine collaboration in computer assisted text refinement

**DOI:** 10.1038/s41598-025-10085-z

**Published:** 2025-07-07

**Authors:** Yicheng Sun, Yi Wang, Hanbo Yang, Richard Suen

**Affiliations:** 1 School of Mechanical and Precision Instrument Engineering, Xi’an University of Technology, Xi’an, China; 2 Faculty of Management, Shenzhen MSU-BIT University, Shenzhen, China

**Keywords:** Natural language process, Human–machine collaboration, Text refinement, Infilling objective, Paraphrase objective, Engineering, Information technology

## Abstract

Human writing often exhibits a range of styles and levels of sophistication. However, automated text generation systems typically lack the nuanced understanding required to produce refined and elegant prose. Due to the inherent one-to-many relationship between inputs and outputs in natural language generation tasks, achieving annotator consistency is challenging. This complexity makes the annotation process considerably more difficult compared to tasks focused on natural language understanding. Our study focuses on the typical task of text refinement, which faces annotation difficulties, aiming to generate sentences with more elegant expressions while preserving the original semantics of the input sentence. This paper proposes a semi-automatic data construction method that combines auto-generation with human judgment. Initially, this method translates collected sentences containing elegant expressions into ordinary expressions through back translation. Subsequently, in an iterative quality control process, data filtering and human judgment are introduced to screen the auto-generated data based on quality standards, resulting in a large-scale text refinement dataset. By replacing manual annotation with human judgment and involving only a small amount of data for human judgment in each iteration, this method significantly reduces annotation difficulty and workload. With minimal human effort, it acquires a substantial amount of labeled data for text refinement, laying a foundation for further research in the field.

## Introduction

The rapid advancement of natural language processing (NLP)^1^ has paved the way for numerous applications that significantly improve human-computer interactions. In its early stages, NLP heavily relied on heuristic rule-based models, where linguists manually crafted extensive rules grounded in linguistic knowledge^2–4^. However, the inherent complexity and evolving nature of human language made it difficult for these predefined rules to comprehensively capture all possible grammatical structures and variations^5^. Among the many emerging applications of NLP, one of the most promising is text refinement, which aims to enhance the elegance and readability of a text while maintaining its original intent^6^. This process plays a crucial role in various fields^7^, including automated content creation, intelligent writing assistants, and machine translation^8^. Despite its importance, the challenge of refining text to achieve a more polished and sophisticated output remains relatively underexplored in current research^9^.

Intelligent Writing Assistance^10^ is an application of NLP, which not only helps writers analyze text for grammatical or stylistic errors but also assists in constructing article outlines and plots from a broader perspective, and even auto-generates content^11^. Due to its ability to expedite the writing process, significant progress has been made in recent years^12–15^. For example, automatic grammar correction can detect and rectify grammatical errors in written sentences^16,17^. Leveraging powerful bidirectional pretrained models like BERT^18^, modern grammar correction systems have greatly improved performance^19^. This enhancement enables online grammar correction services, such as Grammarly, to offer higher-quality services, leading to rapid user base expansion^20^. Rapidly evolving language models have demonstrated promising prospects in automated writing, where, for instance, given a prompt, GPT can generate a coherent article^21^. The proliferation of large-scale pretrained language models, such as GPT^22^ and BERT^18^, has significantly improved the capabilities of NLP systems^23^. These models have demonstrated remarkable performance in various tasks, including text generation, summarization, and translation^24^. However, while they excel at generating coherent and contextually appropriate text, they often fall short in producing refined and elegant prose^25^. This limitation underscores the need for specialized approaches that can enhance the quality of generated text, making it more polished and sophisticated.

Text refinement is a critical component of modern Intelligent Writing Assistance systems, yet there is limited research on it in the existing literature^6^. The goal of text refinement tasks is to generate new sentences corresponding to input sentences, with the new sentences expressing more elegance while maintaining semantic consistency with the original sentences^26^. Text refinement tasks differ from other text rewriting tasks (e.g., text paraphrasing and text style transformation)^27^. In contrast to text paraphrasing, which simply requires the output text to be semantically equivalent to the input text without expanding scenarios or adding substantial text, text refinement demands generating text that is more elegant on top of semantic equivalence and may introduce new elements. For instance, consider the sentence: *“The meeting was good and everyone seemed to enjoy it.”* A paraphrase of this sentence could be: *“The meeting was enjoyable and well-received by all.”* Both sentences convey the same meaning, but no additional elements or elegance are introduced in the paraphrase. In contrast, a refined version might be: *“The meeting was exceptionally engaging, with all participants thoroughly enjoying the discussions.”* Here, the sentence is not only semantically equivalent but also more elegant and introduces a greater sense of engagement.

Text style transformation controls certain attributes in generated text while retaining the original content^28^, such as politeness, emotion, or humor. However, altering styles can change the original intent. For example, consider the sentence *“We regret to inform you that your application has been rejected.”* A style transformation that shifts the tone to be more empathetic might change it to: *“We are sorry to inform you that we were unable to accept your application at this time.”* Although the core content remains the same, the shift in tone introduces a softer, more compassionate style. In contrast, text refinement would focus on enhancing the elegance of the sentence while maintaining the formal, respectful tone and the same core meaning. A refined version of the same sentence might be: *“It is with great regret that we let you know that your application has not been successful.”* The meaning remains intact, but the refinement elevates the formality and sophistication without altering the tone or style as dramatically as in text style transformation^29^. Therefore, compared to other text rewriting tasks, text refinement poses greater difficulty. The process of text refinement involves multiple dimensions, including syntactic correctness^30^, stylistic elegance^31^, and semantic consistency^32^. Achieving this requires a nuanced understanding of language, which can be challenging for automated systems^33^. Human writers excel in these areas due to their deep understanding of language and context^34^. Therefore, integrating human expertise with machine learning techniques through a collaborative approach holds great promise for advancing the field of text refinement.

To address the difficulty of manual annotation in text refinement data, we propose a universal method that combines auto-generation with human judgment to construct a text refinement dataset. This method comprises three steps: (i) collecting sentences with elegant expressions, (ii) using back-translation to generate sentences with ordinary expressions, and (iii) conducting quality control through data filtering and human judgment. By introducing human judgment instead of manual annotation and employing sampling strategies in the iterative process of quality control, this method significantly reduces the difficulty and workload of data annotation. The final text refinement dataset consists of 72,726 auto-generated training data and 4,500 manually evaluated test data. Notably, we constructed the data in English as it is the most widely used and internationally recognized language; however, the proposed method is language-independent and can be extended to other languages such as Chinese, Japanese, Russian, etc.

**Fig. 1:**
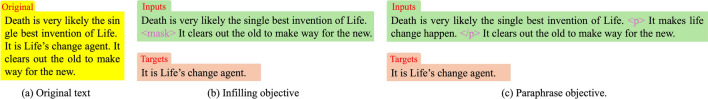
Overview of the two Seq2Seq model training objectives for the text refinement task: (**a**) Original text, (**b**) infilling objective, (**c**) paraphrase objective.

Specifically, we formalize the text refinement task as a sequence-to-sequence text generation task and train the text refinement model using fill-in-the-blank (Infilling) and paraphrasing (Paraphrase) objectives, as shown in Fig. 1. Inspired by these two training objectives, we further introduce pretraining objectives based on different semantic units (words, phrases, and sentences) for infilling (Infilling-style) and paraphrasing (Paraphrase-style) forms. We pretrained a series of baseline models based on the Transformer architecture using these objectives on a large English corpus. Extensive experiments were conducted with these baseline models on the created text refinement dataset, yielding the following main conclusions:

- Processing the text refinement task in a fill-in-the-blank manner (where input includes only context, and the sentence to be refined is treated as blank) performs worse than handling it through paraphrasing because paraphrasing requires inputting both the sentence to be refined and the context simultaneously, providing more information.
- Pretraining with fill-in-the-blank objectives followed by fine-tuning on the text refinement data outperforms directly training on the text refinement data in most evaluation metrics, while pretraining with paraphrasing objectives does not yield further improvements.
- Pretraining objectives containing relatively complete semantic units (phrases and sentences) generally perform better than word-level semantic units.
- Human evaluations demonstrate that our proposed method can generate sentences that are semantically consistent with the original sentence and express more elegance.

In summary, the contributions of this paper can be summarized in three-fold:

- We Introduce a context-aware text refinement task where the goal is to rewrite sentences using more elegant expressions while retaining the original meaning.
- We Propose a semi-automatic data labeling method to address the difficulty of manual annotation in text refinement data and construct a dataset for training and evaluating text refinement systems.
- We Introduce a series of self-supervised pretraining objectives from target forms and semantic units dimensions and use pretrained models trained on a large-scale English corpus as baseline models. Extensive experiments with these baseline models can serve as benchmarks for further research in text refinement.

The remainder of this paper is organized as follows. “Background” presents the background of the Text Refinement Task. “Methodology” elaborates on the proposed approach, including the construction of an elegant expression dataset, generation of ordinary expressions, quality control, and data statistics. “Experimental setup” and “Results and discussion” discuss the experimental design and results. Finally, “Conclusion” summarizes the paper.

## Background

### Contextual text refinement task

Given a set of input texts: $\{C_{left}, S_{ordinary}, C_{right}\}$, where $S_{ordinary}$ is an ordinary expression sentence, and $C_{left}$ and $C_{right}$ are the left and right contexts of $S_{ordinary}$, the text refinement task aims to refine $S_{ordinary}$ to achieve a more elegant expression, $S_{polished}$. The refined text $\{C_{left}, S_{polished}, C_{right}\}$ should be contextually coherent and semantically consistent with the original text. We formalize the text refinement task as a sequence-to-sequence text generation task^35^. Specifically, given the input sequence *X* = $\{x_0,..., x_N\}$ and output sequence *Y* = $\{y_0,..., y_T\}$, the conditional probability of the output sequence *Y* is as follows:

1 $$\begin{aligned} p(Y | X) = \prod _{i=1}^{T} p(y_t | y_{<t}, X) \end{aligned}$$

As shown in Fig. 1, there are two ways to construct the input sequence *X* and output sequence *Y* for the training objectives of the Seq2Seq model for the text refinement task.

Infilling objective: As depicted in Fig. 1b, this objective uses a special placeholder $\langle mask \rangle$ to mask the sentence in the input sequence that needs refinement. The model predicts the refined sentence based on the context of the masked sentence. Formally, the input sequence *X* and output sequence *Y* of the Seq2Seq model are represented as:

2 $$\begin{aligned} \begin{aligned} X&= \{ C_{left}, \langle mask \rangle , C_{right} \} \\ Y&= S_{polished} \end{aligned} \end{aligned}$$

Paraphrase objective: Illustrated in Fig. 1c, this objective utilizes two special placeholders $\langle p \rangle$ and $\langle /p \rangle$, with the text between them being the sentence in the input sequence requiring refinement. The model predicts the refined sentence based on the sentence itself and its context. Formally, the input sequence *X* and output sequence *Y* of the Seq2Seq model are represented as:

3 $$\begin{aligned} \begin{aligned} X&= \{ C_{left}, \langle p \rangle S_{ordinary} \langle /p \rangle , C_{right} \} \\ Y&= S_{polished} \end{aligned} \end{aligned}$$

### Semi-automatic data labeling method

Research on text refinement is currently limited, primarily due to the significant difficulty in manually annotating text refinement data^36^. The traditional process of manually labeling text refinement data involves providing a sentence to annotators and requesting them to rewrite the sentence into a more elegant expression^37^. This process faces the following challenges:

- Defining specific elegance in writing is challenging. Elegant expressions can involve various writing techniques such as the appropriate use of rhetorical devices, diverse sentence structures, or references to famous quotes.
- Judging elegance is highly subjective. Individuals with different cultural backgrounds, educational levels, or aesthetic tastes may have varying opinions on whether a sentence is elegant, leading to significant discrepancies among annotators and low consistency.
- Rewriting sentences elegantly requires a high level of expertise, demanding annotators with literary skills and aesthetic abilities, which many existing annotators may lack.

**Fig. 2:**
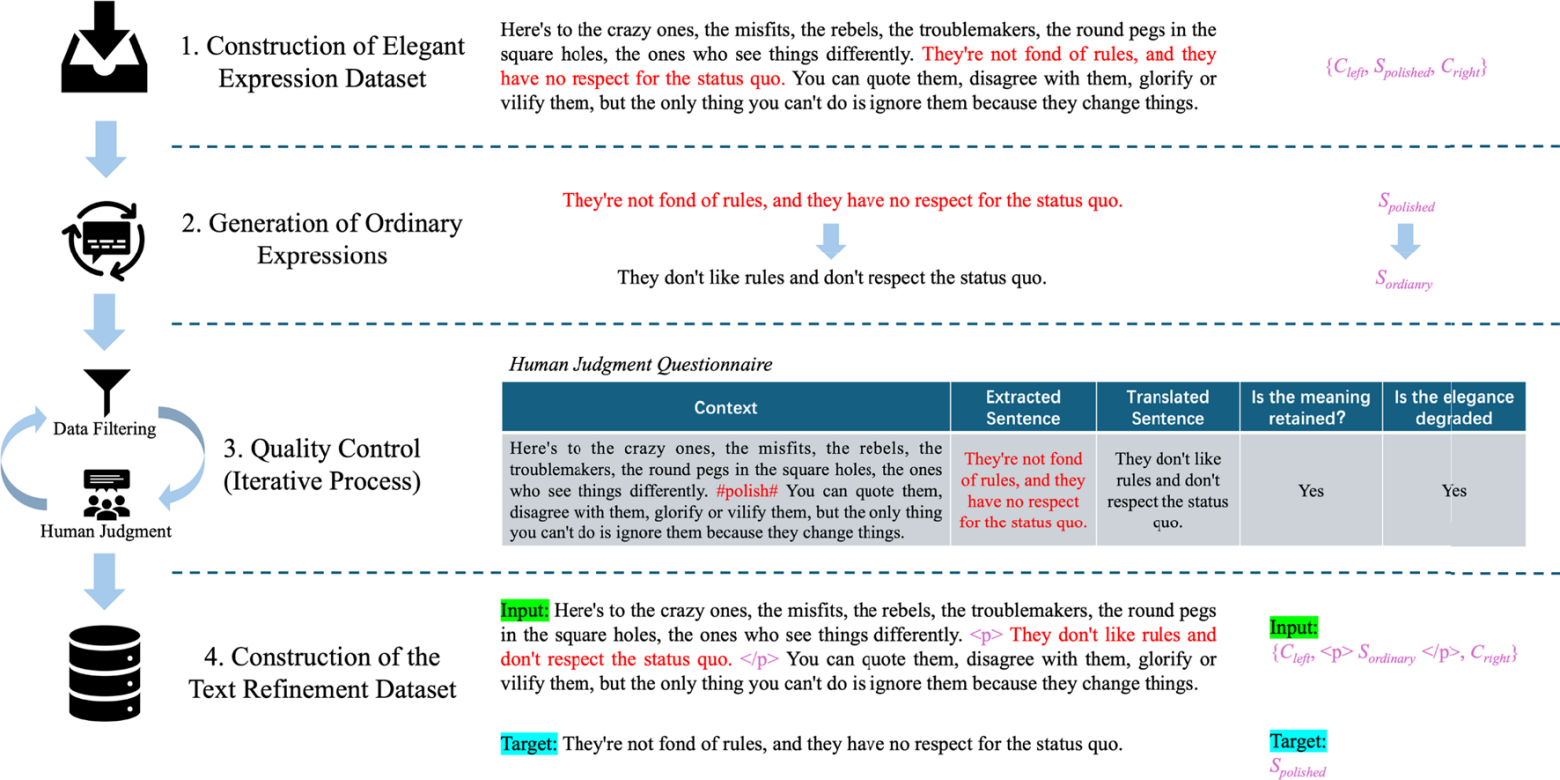
Schematic representation of the semi-automatic data construction method. **Note:** The right half of the figure provides examples of text transformations at each step along with formal representations. (i) Collect sentences containing elegant expressions and their contexts. (ii) Utilize back-translation to transform these sentences into ones with ordinary expressions. (iii) Ensure the generated sentences maintain the original meaning while reducing the level of elegance through quality control. (iv) Concatenate the compliant sentences and their contexts to create a source for a text refinement case, with the original sentence serving as the target for the case (illustrated here using the paraphrase objective as an example).

Overall, it is evident that acquiring high-quality refinement data through traditional manual annotation methods is challenging. However, it is noteworthy that determining which of two sentences, conveying the same meaning but expressed differently, is much easier than completely rewriting a sentence to make it more elegant. Based on this fact, we propose a semi-automatic data construction method that combines auto-generation with human judgment. As shown in Figure 2, this method primarily involves three steps:

(i) Collecting well-known and elegantly expressed sentences.
(ii) Automatically transforming these elegantly expressed sentences into ordinary expressions with the same meaning.
(iii) Ensuring the generated data meets specific standards through quality control.

The first two steps involve two sentences—one elegant, sourced from our manually curated data, and one ordinary, obtained through back-translation—while the final step uses human judgment to ensure the quality of the generated data. To ensure experimental validity, we introduce human judgment (selecting the more elegant expression between two sentences) in the final step instead of manual annotation (rewriting sentences to express them more elegantly), significantly reducing the requirements for annotators. By sampling a small amount of data for human judgment, substantial amounts of compliant data can be obtained at a lower labor cost. We will elaborate on the roles of each part in Fig. 2 in “Methodology”.

In accordance with the aforementioned semi-automatic data construction method, this paper illustrates the process of constructing an English text refinement dataset using idioms as an example. “Construction of elegant expression dataset” provides the rationale for selecting idioms as elegant expressions and the source of idiom data. “Generation of ordinary expressions” outlines the idea and method of using back-translation to transform sentences with elegant expressions into sentences with common expressions. “Quality control” elaborates on the quality control process, focusing on data filtering and manual evaluation as two iterative sub-steps.

## Methodology

### Construction of elegant expression dataset

We investigated the top 10% ranking books on the Amazon bestseller list from 2020 to 2024 and downloaded electronic versions of some of these books from the Zlibrary. We extracted elegant text excerpts from each book. To enhance the dataset construction process, we enlisted the help of three doctoral students with over five years of literary experience, resulting in 50,000 sentences, which form Dataset 1. Each sentence paragraph consists of at least three sentences, with the middle sentence being $S_{polished}$, consistent with the format of the refined text $\{C_{left}, S_{polished}, C_{right}\}$ as defined in “Contextual text refinement task”.

The sentences in Dataset 1 primarily originate from online e-books within a relatively narrow domain. To further expand the domains covered by the refinement dataset, we collected partial sentences from the United Nations Parallel Corpus (UN6)^38^, constituting Dataset 2. This corpus comprises official UN records and other meeting documents, offered in six official UN languages and aligned at the sentence level. We selected a portion of English sentences from the UN documents, managed by two doctoral students, retrieving a total of 40,000 English sentences as $S_{polished}$. The $C_{left}$ and $C_{right}$ were identified based on the index of each sentence in the corpus. Table 1 provides an overview of these two datasets.

### Generation of ordinary expressions

After collecting elegant expression sentences, the next step involves transforming them into ordinary expressions. Machine translation models trained on large-scale data tend to generate ordinary expressions encountered during their pretraining process due to the differences in expression and content of each sentence in the data. However, after reviewing the literature^39,40^, we found a more suitable approach: translating English sentences into Chinese and then translating the generated Chinese sentences back into English, resulting in sentences with similar meanings but significantly reduced elegance^41^. This method, known as back-translation^42^, is commonly used for data augmentation^43^.

Back-translation is widely applied in various natural language processing tasks^44^. For instance, in text style transformation tasks, Prabhumoye et al.^45^ have shown that translating sentences from the source language to the target language leads to translated sentences that retain the meaning of the source sentences but do not preserve the original author’s unique writing style^46^. Similarly, back-translation is utilized in paraphrase generation tasks to produce multiple candidate interpretations^47^.

We utilized the back-translation method to automatically generate sentences for constructing the text refinement dataset. Specifically, we employed Google’s translation service to translate the $S_{polished}$ part of the English sentences using the aforementioned two-step back-translation method to obtain ordinary expression sentences, $S_{ordinary}$. Since the United Nations corpus provides human-translated Chinese sentences corresponding to English sentences, we simply called the translation service to translate all Chinese sentences back into English, extracting the $S_{ordinary}$ part corresponding to the originally English sentences’ $S_{polished}$ section, thereby obtaining these English sentences’ ordinary expressions.

### Quality control

After obtaining ordinary expressions with reduced elegance in the previous step, it is essential to ensure data quality—i.e., to verify that the sentences obtained through back-translation have the same meaning as the original sentences but with reduced elegance. The final step of dataset construction includes a quality control process. Quality control involves two sub-steps: data filtering and human judgment. The former filters the data by rejecting defective samples, while the latter samples a portion of the filtered data for manual evaluation to determine if it meets set criteria. It is crucial to note that the quality control process is iterative, as depicted in Step Three in Fig. 2. The data filtering step initially filters the data according to predetermined criteria, and the human judgment step assesses whether the filtered data meets predefined standards. If the standards are met, the quality control process ends; if not, the data filtering parameters are adjusted, and the process repeats until the sampled data post-human judgment reaches the set criteria.

#### Data filtering

We filter the data obtained in the previous section based on the following aspects (notably, the parameters provided in each item represent the final valid values obtained after multiple rounds of iteration in the quality control process):

- If the translated sentences have missing parts or additions–mainly due to translation software inaccuracies in understanding sentence meanings–we directly discard such flawed samples.
- To reduce the difficulty of the refinement task and retain information, only English sentences with lengths between 30 and 130 words are retained.
- Due to highly uneven sentence lengths between punctuations in corpus sentences, some sentences might result in excessively long or short Context and Right sections. To avoid an imbalanced dataset, this data subset is reduced, accounting for no more than 10% of the total dataset.
- Some sentences might employ obscure expressions such as allusions, dialects, or nursery rhymes influenced by regional and cultural differences across countries. To mitigate this imbalance, data from these subsets is trimmed, comprising no more than 5% of the total dataset.

#### Human judgment

**Fig. 3:**
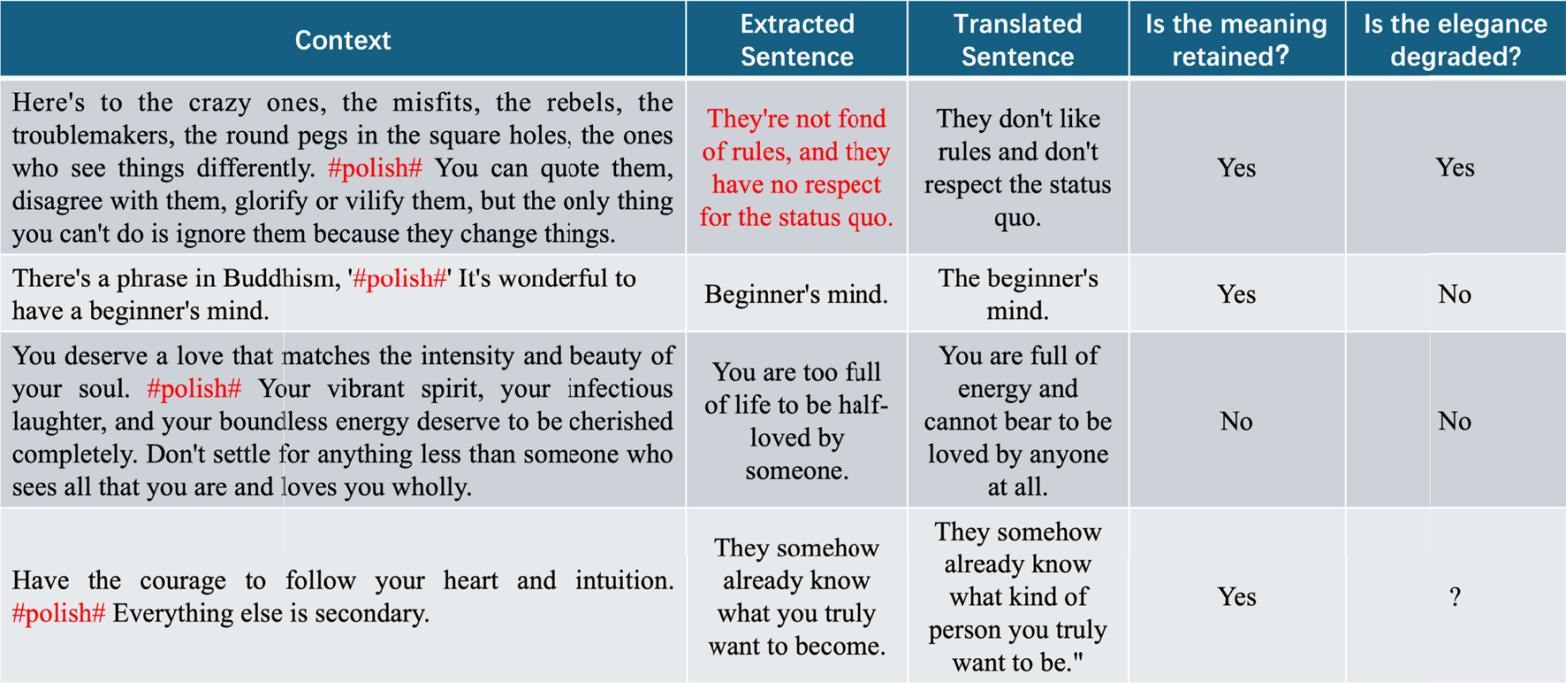
Questionnaire form for human judgment. The last two columns in the table contain the questions that need to be answered.

To ensure that the sentences generated using the back-translation method have the same meaning as the original sentences but with reduced elegance, human judgment is employed to assess the quality of the data obtained through data filtering. Specifically, this evaluation process utilizes a questionnaire table as depicted in Fig. 3, where each row represents a test case. The “Context” column provides the context of the sentence requiring refinement ($C_{left}$ and $C_{right}$) along with the placeholder “$\#polish\#$” representing the sentence that needs refinement; the “Polish” column offers the extracted $S_{polished}$ sentence from the original excerpt; and the “Back-translated” column presents the sentence $S_{ordinary}$ derived using the back-translation method from $S_{polished}$.

When evaluating each instance in the table, the evaluator replaces the placeholders marked with “$\#polish\#$” in the context with the sentences from the second and third columns and answers the following two questions:

(i) Does the back-translated sentence have the same meaning as the original sentence? (Is the meaning retained?)
(ii) Is the elegance level of the back-translated sentence lower than that of the original sentence? (Is the elegance degraded?)

Cases where both questions are answered “Yes” represent instances that meet the refinement requirements, indicating that $S_{polished}$ and $S_{ordinary}$ have the same meaning but with improved elegance.

We enlisted 10 professional experts in the field of linguistics as evaluators. In each iteration of the quality control process, 100 randomly sampled entries from the data filtered in the previous round were provided to the evaluators for manual assessment. The iterative process continues until 85% of the sampled cases meet the refinement criteria. While theoretically, higher quality refinement data could be obtained through further iterations, considering manpower and time constraints, we did not pursue stricter quality standards. It is worth noting that, unlike the relatively lenient quality standards used during the iterative process, more stringent criteria were applied during manual assessment to construct the test set. Specifically, all cases in the test set must strictly adhere to the text refinement requirements.

### Data statistics

The statistical information of the text refinement task dataset we obtained is presented in Table 1. The text refinement dataset, data-ebook, is divided as follows: In the cases obtained through the three steps of data construction in Dataset 1, 2500 instances were labeled using the human judgment method from the previous section to form the test set. Following this, 10,000 instances were randomly sampled for the validation set, with the remaining instances allocated to the training set. Similarly, from the cases obtained through the aforementioned steps in UN6, 2000 instances were manually labeled as the test set of data-UN6.

**Table 1:** Statistics of the text refinement dataset.

Dataset	Training set	Validation set	Test set	Avg. length ($S_{polished}$)
data-ebook	35,785	10,000	2500	72
data-UN6	36,941	–	2000	89

To assess the quality of the dataset, the validation set of data-ebook was sampled five times using the method outlined in “Human judgment”. The average proportion (standard deviation) of cases meeting the refinement requirements across the five samples is 86.2% (2.47), indicating that the final refinement dataset has essentially reached the preset quality standards.

## Experimental setup

Due to the outstanding performance of pretrained models in various natural language processing tasks, we employ pretrained models as the baseline models for the text refinement task. Pretrained models typically utilize self-supervised learning tasks like Masked Language Model (MLM)^18^ or Denoising Autoencoder (DAE)^48^ for training. While the self-supervised learning tasks during the pretraining phase may differ from the supervised learning tasks during fine-tuning^49^, if the training objectives in the pretraining phase are similar to those in the fine-tuning phase, the knowledge learned by the model during pretraining can more easily transfer to downstream tasks^50^. Based on the two training objectives (Infilling Objective and Paraphrase Objective) proposed for the text refinement task in “Contextual text refinement task”, we introduce two types of task-specific pretraining objectives: *Infilling-style Pre-training Objective* and *Paraphrase-style Pre-training Objective*.

### Infilling-style pre-training objective

The Infilling training objective used to train Seq2Seq text refinement models (Fig. 1b is akin to the Span-corruption pretraining objective proposed by Raffel^51^. This objective involves replacing a randomly selected token span in the input sequence with mask tokens and predicting the masked token span. Inspired by this, we modify the Span-corruption objective to serve as the training objective for filling-style pretraining of text refinement task models.

**Fig. 4:**
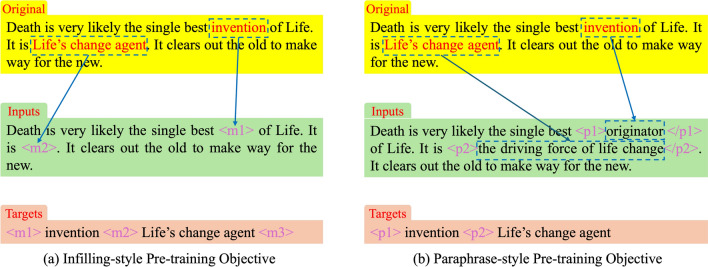
The two types of pretraining objectives for the text refinement task: (**a**) infilling-style pre-training objective, (**b**) paraphrase-style pre-training objective.

The process of constructing training samples using the Infilling-style objective is illustrated in Fig. 4a. In this setup, the input sequence is constructed as follows: randomly select several words from the original text and replace them with sentinel tokens (e.g., $\langle m1 \rangle$ and $\langle m2 \rangle$ in the example). It’s important to note that each sentinel token in a sample is unique, and consecutive words are replaced by a single sentinel token. For example, “Life’s change” and “agent” are contiguous in the sentence, so they are replaced by a single sentinel token $\langle m2 \rangle$. The output sequence comprises the token spans replaced in the input sequence, with each span prefixed by the sentinel token that replaced it in the input sequence and an additional sentinel token at the end of the last span (e.g., $\langle m3 \rangle$ in the example).

In literature^52^, BERT models were pretrained using the Whole Word Masking (WWM) strategy, which masks phrases rather than individual words to allow the model to learn word boundary information. Drawing inspiration from this, we considered three different semantic units - words, phrases, and sentences - when selecting the scope of masked tokens. This approach enables the model to learn semantic information at various granularity levels. Individual words are masked at the level of single English words. Phrases, typically comprising two or more words, are masked at the phrase level. Sentences, forming a complete semantic unit, are first divided into multiple sentences based on common punctuation marks (“.”, “; ”, “, ”, “?”, “!”). Following this, segments of random contiguous sentences are selected for masking replacement based on a Poisson distribution ($\lambda$ = 5), ensuring that the total number of masked characters in all selected sentences does not exceed 15% of the total characters in the original text.

### Paraphrase-style pre-training objective

MacBERT^53^, during the training of the BERT Masked Language Model task, replaced masked tokens with similar words to bridge the gap between pretraining and fine-tuning learning objectives^54^. Inspired by this concept, we propose a paraphrase-style training objective for pretraining the text refinement model. The paraphrase-style training objective involves replacing masked text segments with their paraphrased meanings instead of mask tokens as done in filling-style training objectives to construct the input sequence for the pretraining model. Unlike MacBERT’s method that relies on the Encoder-only structure of the BERT model, this model structure ensures a one-to-one correspondence between inputs and outputs.

The process of constructing training samples using the paraphrase-style objective is illustrated in Fig. 4b. Taking the example sentence in the figure, words “Life’s change” and the phrase “agent” are randomly selected for replacement. They are then substituted with their respective paraphrased meanings “originator” and “the driving force of life change” in the constructed input sequence. To enable the model to learn boundary information of the replaced text segments, special tokens $\langle pi \rangle$ and $\langle /pi \rangle$ denote the start and end of each paraphrased segment in the input sequence, resulting in the final input sequence “Death is very likely the single best $\langle p1 \rangle$originator$\langle /p1 \rangle$> of Life. It is $\langle p2 \rangle$the driving force of life change$\langle /p2 \rangle$. It clears out the old to make way for the new.”. The construction of the output sequence is similar to how output sequences are created in filling-style pretraining objectives. The replaced text segments are separated by $\langle pi \rangle$ markers in the output sequence, representing the initial positions of the paraphrased sections in the original text, yielding the output sequence “$\langle p1 \rangle$invention $\langle p2 \rangle$Life’s change agent”. Considering the significant variability in the meaning of individual words based on context, it is challenging to provide paraphrases for single words. Hence, for the paraphrase-style training objective, only phrases and sentences are considered as semantic units for paraphrasing purposes.

## Results and discussion

### Evaluation metrics

In this section, the text refinement task is formalized as a natural language generation task. Due to the complexity of natural language, evaluating language generation is a challenging task. It is widely acknowledged that each evaluation method can only capture certain aspects of language generation quality. A comprehensive assessment of a language generation model often requires multiple evaluation methods and metrics to draw reliable conclusions. Therefore, we employ various evaluation methods to assess the text refinement task comprehensively, aiming to evaluate the model’s performance from different perspectives.

#### Vector similarity-based methods

Text refinement requires the refined text to convey a similar meaning to the original text. While the generated text may retain the same meaning as the original, it might use different words compared to the reference text. Evaluation metrics based on vector similarity calculate cosine similarity between vector representations of two texts, providing a soft measure of similarity^55^. We utilize three word embedding-based metrics to evaluate the similarity between the generated refined text and the reference text^56^. These metrics differ in how they calculate sentence vectors using word embeddings^57^ to measure the similarity^58^ between two sentences.

Embedding average (EA): The EA metric first calculates the average of word vectors composing the reference sequence and the generated sequence to obtain sentence vectors. It then computes the cosine similarity between these two sentence vectors to derive the EA score. The formula for EA is given by:

4 $$\begin{aligned} EA(x, \hat{x}) = \text {cos}\_\text {sim}\left( \frac{1}{|x|} \sum _{i=1}^{|x|} {\bf{w}}_i^x, \frac{1}{|\hat{x}|} \sum _{j=1}^{|\hat{x}|} {\bf{w}}_j^{\hat{x}}\right) \end{aligned}$$

where ${\bf{w}}_i^x$ and ${\bf{w}}_j^{\hat{x}}$ represent the word vectors of the reference sequence *x* and the generated sequence $\hat{x}$, respectively. The $\text {cos\_sim}$ function calculates the cosine similarity between the average vectors of the reference and generated sequences.

Greedy matching (GM): The GM metric calculates the one-way greedy matching score between two sequences. For example, the greedy matching score $G(x, \hat{x})$ from reference sequence *x* to generated sequence $\hat{x}$ is computed as follows:

5 $$\begin{aligned} G(x, \hat{x}) = \frac{1}{|x|} \sum _{i=1}^{|x|} \max _{j \in [1, |\hat{x}|]} \text {cos}\_\text {sim}(x_i, \hat{x}_j) \end{aligned}$$

where $\text {cos\_sim}$ calculates the similarity between two word vectors. The one-way greedy matching score $G(\hat{x}, x)$ from generated sequence $\hat{x}$ to reference sequence *x* is computed similarly. The final GM score averages these scores in both directions:

6 $$\begin{aligned} GM(x, \hat{x}) = \frac{1}{2} \left( G(x, \hat{x}) + G(\hat{x}, x) \right) \end{aligned}$$

Vector extrema (VE): The VE metric first computes sentence vectors, with each dimension of a vector taking the extremum values of the corresponding dimensions of all word vectors composing the sentence:

7 $$\begin{aligned} e_d^x = \text {ext}\left( \{ w_{d,i}^x \}\right) \end{aligned}$$

where $e_d^x$ represents the value of dimension *d* of the sentence vector $e^x$. The right side of the equation indicates that when the absolute value of the negative extremum is greater than the positive extremum, the value of the sentence vector in that dimension is set to the negative extremum. The final VE score still calculates the cosine similarity between the two sentence vectors:

8 $$\begin{aligned} VE(x, \hat{x}) = \text {cos}\_\text {sim}(e^x, e^{\hat{x}}) \end{aligned}$$

In this section, pretrained word embeddings are used to calculate the above three vector similarity-based metrics (EA, GM, VE) between the refined text and the reference text.

#### BERTScore

Since words can have varying semantics in different contexts, static word embedding-based metrics struggle to capture this variability. Hence, researchers have proposed evaluation methods that utilize context-aware word embeddings to compute similarity^22^, such as BERTScore^59^. Apart from the three static word embedding-based metrics mentioned earlier, we also incorporate the BERTScore metric to evaluate the text refinement task.

BERTScore comes in three forms: recall ($R_{\text {BERT}}$), precision ($P_{\text {BERT}}$), and F1 score ($F_{\text {BERT}}$). Recall is calculated by matching each word in *x* with each word in $\hat{x}$, then computing precision, and ultimately the F1 score:

9 $$\begin{aligned} & R_{\text {BERT}} = \frac{1}{|x|} \sum _{i=1}^{|x|} \max _{j \in [1, |\hat{x}|]} \text {cos}\_\text {sim}(x_i, \hat{x}_j) \end{aligned}$$

10 $$\begin{aligned} & P_{\text {BERT}} = \frac{1}{|\hat{x}|} \sum _{j=1}^{|\hat{x}|} \max _{i \in [1, |x|]} \text {cos}\_\text {sim}(\hat{x}_j, x_i) \end{aligned}$$

11 $$\begin{aligned} & F_{\text {BERT}} = \frac{2 \cdot R_{\text {BERT}} \cdot P_{\text {BERT}}}{R_{\text {BERT}} + P_{\text {BERT}}} \end{aligned}$$

In this section, the tool of bert_score is utilized to compute the BERTScore metric between the generated refined text and the reference text.

#### Diversity and generated text length

Furthermore, we assess the diversity of the generated text by computing the ratios of unique unigrams, bigrams, and sentences in the generated refined text over the total number^60^, denoted as Dist-1, Dist-2, and Dist-S. Higher values of diversity metrics indicate better text diversity.

### Results comparison

#### Model performance evaluation

Prior to the experiments, we conducted pretraining and fine-tuning. The goal of pretraining^61^ and fine-tuning^62^ is to enable the model to excel in a wide range of language tasks. Pretraining imparts basic language understanding and generation capabilities to the model by learning language patterns and knowledge from large-scale text data^63^. Fine-tuning further adjusts the model on specific task or domain data to enhance its performance in these particular application scenarios^64^. We selected additional data from electronic books for pretraining, which is not included in the data-ebook and data-UN6 datasets. The training data for fine-tuning was created using the dataset data-ebook proposed in Table 1.

For our experiments, we employed three different Text Polishing Pre-trained Models (TPPMs): T5, BART, and GPT-3. The TPPM is a model designed to enhance text quality by predicting missing spans of text, with a focus on improving fluency and coherence in generated text. T5 is a versatile pre-trained model that can handle a wide range of NLP tasks, including text refinement and infilling, which aligns well with the objectives of TPPM. BART, which combines a bidirectional encoder (like BERT) and an autoregressive decoder (like GPT), was chosen for its robust performance in text generation tasks, such as summarization and text restoration. Finally, GPT-3, known for its powerful generative capabilities, was included to test how autoregressive models perform in infilling-style objectives, given its ability to generate coherent text from partial information. Together, these models allow us to compare different architectures and their suitability for the text polishing task.

We compared the two training objectives for the text refinement task. Table 2 presents the experimental results of the TPPMs, which were fine-tuned using two distinct objectives: the infilling objective and the paraphrase objective. The experiments were conducted on separate test sets from data-ebook and data-UN6, as well as a combined test set (data-ebook+UN6), which includes data from both sources.

**Table 2:** Experimental results of models with different training objectives.

Dataset	Model	Training objective	Contextual similarity			BERTScore			Diversity		
			EA	GM	VE	P	R	F1	Dist-1	Dist-2	Dist-S
data-ebook	T5	Infilling objective	0.732	0.291	0.573	0.628	0.610	0.619	**0.128**	0.603	0.968
		Paraphrase objective	**0.899**	**0.425**	**0.852**	**0.891**	**0.815**	**0.851**	0.126	**0.679**	**0.992**
	BART	Infilling objective	0.764	0.315	0.592	0.630	0.622	0.626	0.131	0.608	0.974
		Paraphrase objective	**0.820**	**0.403**	**0.817**	**0.838**	**0.794**	**0.814**	**0.133**	**0.659**	**0.971**
	GPT-3	Infilling objective	0.725	0.288	0.557	0.621	0.603	0.612	**0.126**	0.600	0.954
		Paraphrase objective	**0.862**	**0.417**	**0.838**	**0.879**	**0.803**	**0.838**	0.124	**0.668**	**0.985**
data-UN6	T5	Infilling objective	0.706	0.282	0.598	0.636	0.618	0.626	0.114	0.589	0.975
		Paraphrase objective	**0.844**	**0.396**	**0.871**	**0.894**	**0.859**	**0.876**	**0.115**	**0.666**	**0.996**
	BART	Infilling objective	0.709	0.288	0.603	0.642	0.629	0.636	**0.115**	0.597	0.974
		Paraphrase objective	**0.803**	**0.371**	**0.809**	**0.842**	**0.830**	**0.836**	**0.115**	**0.649**	**0.980**
	GPT-3	Infilling objective	0.692	0.278	0.586	0.629	0.611	0.620	0.113	0.572	0.971
		Paraphrase objective	**0.828**	**0.366**	**0.851**	**0.870**	**0.851**	**0.858**	**0.114**	**0.659**	**0.990**
data-ebook+UN6	T5	Infilling objective	0.698	0.278	0.558	0.621	0.608	0.614	**0.107**	0.576	0.947
		Paraphrase objective	**0.825**	**0.375**	**0.833**	**0.873**	**0.798**	**0.834**	**0.107**	**0.626**	**0.985**
	BART	Infilling objective	0.702	0.281	0.563	0.629	0.612	0.622	**0.109**	0.582	0.951
		Paraphrase objective	**0.810**	**0.358**	**0.825**	**0.859**	**0.763**	**0.808**	0.108	**0.617**	**0.971**
	GPT-3	Infilling objective	0.690	0.273	0.549	0.619	0.601	0.610	0.106	0.570	0.939
		Paraphrase objective	**0.821**	**0.364**	**0.829**	**0.867**	**0.774**	**0.818**	**0.107**	**0.622**	**0.982**

We highlight the highest score in each column in bold for the same model and dataset. The following abbreviations are used for metrics: EA stands for embedding average, GM for greedy matching, VE for vector extrema, P for precision, R for recall, and F for F1 score.

From the results in Table 2, it is evident that the model fine-tuned with the paraphrase objective outperforms the one fine-tuned with the infilling objective across almost all evaluation metrics. Specifically, the performance on the individual data-ebook and data-UN6 datasets excels over the combined data-ebook+UN6 dataset. This is because, as the amount of data increases and diverse data sources are introduced, the characteristics of the data vary, which can cause the model’s performance to decrease due to these variations. This is a commonly observed phenomenon in such scenarios.

From the overall performance of the models across the three datasets, T5 achieves the best results, followed by GPT-3, and then BART. An interesting observation is that the T5 model fine-tuned with the paraphrase objective consistently achieves the highest performance across all three datasets, significantly outperforming the other models. This trend is particularly evident in the F1 score, where T5 demonstrates a clear advantage, highlighting its ability to refine text and enhance its elegance while maintaining semantic consistency. The paraphrase objective is well-suited for text refinement tasks, as it focuses on generating linguistically sophisticated and more polished versions of the input text, which aligns closely with our task of improving the quality of text.

However, when fine-tuned with the infilling objective, BART slightly outperforms T5, suggesting that for certain text generation tasks involving more structured or form-based output, BART may have a slight edge. This is particularly relevant in scenarios where more precise, contextually accurate text generation is required, as the infilling objective emphasizes completing or filling in gaps within text. The slight advantage of BART in this case can likely be attributed to its architecture, which is optimized for tasks requiring sequence-to-sequence modeling with bidirectional context, as well as the nature of its training regime, which may make it more robust in certain structured text generation tasks.

**Fig. 5:**
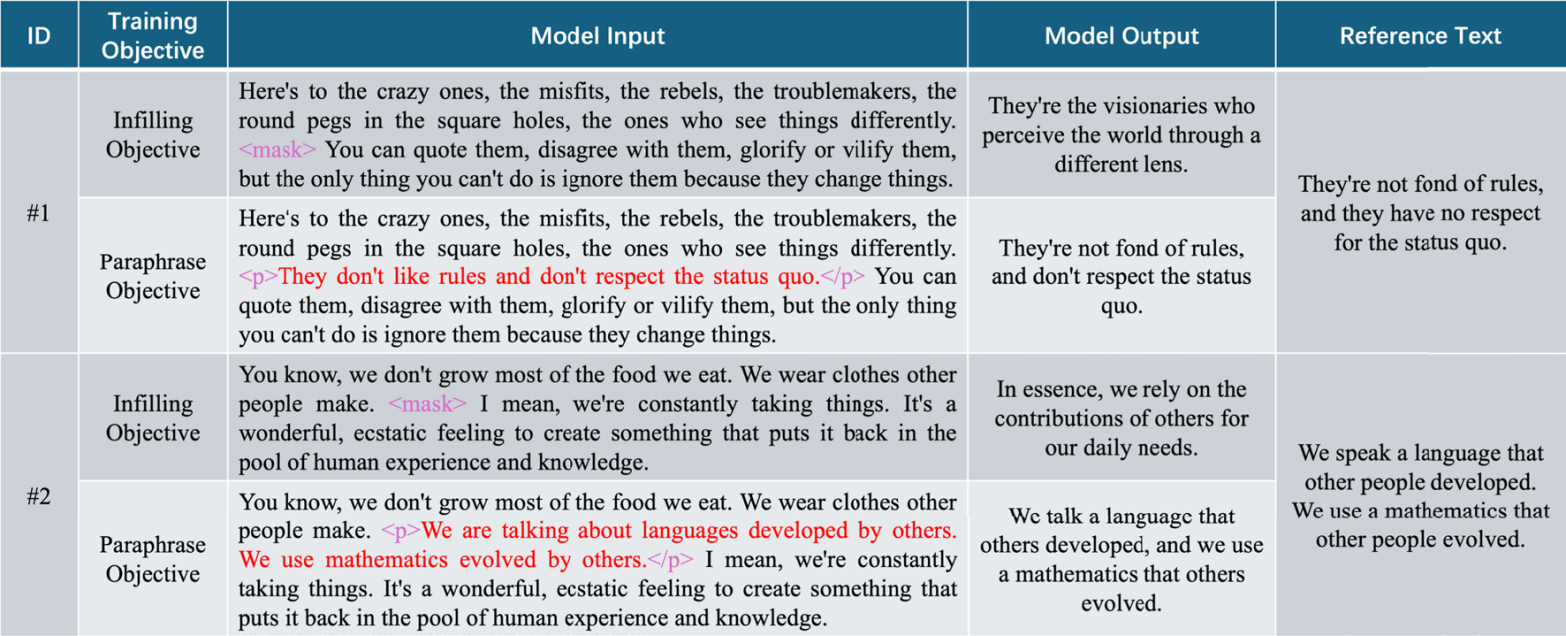
Examples of the model TPPM (O=IS, U=Char) fine-tuned with two training objectives (Infilling and Paraphrase) to refine two paragraphs. For the infilling objective, the model generates a sentence that fits the context around the $\langle mask \rangle$ position in the input text. For the paraphrase objective, the model produces a refined sentence for the segment marked with $\langle p \rangle$ and $\langle /p \rangle$ in the input text.

To further explain this result, Fig. 5 provides two examples of text refinement using models trained with the infilling and paraphrase objectives. By examining the text generated by the model trained with the infilling objective, we notice that although the generated sentences form coherent text with the context, they do not convey the same meaning as the reference text, resulting in significant differences in BERTScore scores between the two objectives. The experimental results indicate that the paraphrase objective is a superior choice for the text refinement task compared to the infilling objective. Therefore, in practical scenarios, employing the paraphrase objective for model fine-tuning can lead to better text refinement results.

#### Elegance evaluation

To address the limitation in the definition and quantification of “elegance” in text refinement, we conducted an experimental evaluation to assess the degree of elegance in the refined texts. We selected the results from the Paraphrase Objective experiments, as it consistently outperformed the Infilling Objective across multiple evaluation metrics. For the selection of the best-performing refined texts, we utilized the Diversity-Promoting Paragraph Sampling (DPP) method, which ensures the selection of more diverse and representative samples from the output texts generated by the T5 model. By applying DPP, we could capture a broader variety of linguistic styles and enhancements in the refined texts, avoiding overrepresentation of similar sentence structures and ensuring a more robust evaluation.

We selected a set of 1200 representative texts from the T5 model’s outputs. These texts were evaluated by four experts, each holding a PhD in literature and possessing over 10 years of experience in teaching and research in the field. Each expert independently assessed the performance of all three versions of each text–the input text, the model-generated output text, and the original text–resulting in a comprehensive evaluation of all 1200 texts. The final scores for each version were then averaged across all four experts to provide a balanced evaluation. The experts were tasked with evaluating these versions based on three key criteria: Linguistic Elegance, Semantic Consistency, and Overall Quality. Detailed scoring guidelines can be found in Table 3.

**Table 3:** Evaluation criteria and scoring guidelines (1–5 scale).

Criterion	Score	Description
Linguistic elegance	5	Highly polished text with exceptional fluency and sophistication; sentence structures and vocabulary are elevated.
	4	Clearly more elegant than the original, with improved fluency and vocabulary; minor improvements still possible.
	3	Some improvements in elegance, but changes are modest or inconsistent; text may still feel flat.
	2	Limited improvements; text remains basic in fluency and style.
	1	Largely unchanged; no noticeable enhancement in fluency or sophistication.
Semantic consistency	5	Meaning fully preserved; may even improve clarity without altering content.
	4	Largely preserves meaning; small wording changes do not affect interpretation.
	3	Most meaning retained; minor distortions or shifts in tone may occur.
	2	Noticeable changes in meaning that could affect interpretation.
	1	Meaning significantly altered or misrepresented; ambiguity or key ideas lost.
Overall quality	5	Highly engaging, well-structured, and coherent; smooth and enjoyable to read.
	4	Clear and coherent with good flow; minor improvements could increase polish.
	3	Understandable but may lack flow or have awkward structure.
	2	Difficult to follow due to issues with coherence, clarity, or structure.
	1	Poorly written and disjointed; major readability and structural problems.

A detailed PDF document outlining the three proposed evaluation metrics is available on our GitHub repository at: https://github.com/Researcher-xaut/text_refinement

**Table 4:** Evaluation of text semantics based on three key metrics.

Text version	Linguistic elegance	Semantic consistency	Overall quality
Input texts	3.27 ± 0.06	4.08 ± 0.05	3.52 ± 0.04
Model output texts	4.49 ± 0.04	4.36 ± 0.04	4.42 ± 0.05
Original texts	4.34 ± 0.04	4.41 ± 0.04	4.49 ± 0.04

The results from this evaluation are presented in Table 4, which illustrates the comparative performance of the input texts, the model-generated output texts, and the original texts across the three evaluation metrics. The data clearly indicate that the model-generated output texts, particularly those refined using the Paraphrase Objective, consistently outperform the input texts across all three evaluation criteria. Notably, the output texts achieved higher ratings for linguistic elegance, highlighting the model’s ability to enhance the overall quality and style of the sentences while preserving their original meaning. The semantic consistency scores further affirm that the model maintained the integrity of the text, ensuring that the refined version stayed true to the original message.

To provide a more thorough understanding of the evaluation, we conducted detailed interviews with four experts. The experts reviewed the texts by comparing three versions: the input, the model-generated output, and the original. Their evaluations revealed interesting nuances between the Model Output Texts and the Original Texts. The model-generated outputs were highly praised for their increased sophistication and improved stylistic qualities, demonstrating the model’s proficiency in refining language. In contrast, while the Original Texts were often more direct and clear, they were also deemed less engaging or sophisticated in terms of linguistic style.

Further analysis of the results reveals that the refined texts, when compared to the original versions, exhibit distinct improvements in linguistic sophistication. The T5 model, fine-tuned with the paraphrase objective, elevates the elegance of the sentences without altering their core meaning. For example, the model refines the sentence: *“He was tired, but he kept walking because he had no choice.”* to: *“Exhausted, he continued walking, knowing he had no other option.”* In this case, the model enhances the fluency and sophistication of the language by introducing more varied vocabulary and a smoother structure. These improvements contribute to a more polished and engaging text, making it better suited for higher-level literary contexts, while still maintaining the original intent.

In conclusion, this evaluation demonstrates that the model’s text refinement process successfully improves the elegance of the text, as measured by linguistic elegance, semantic consistency, and overall quality. The findings show that while the Model Output Texts and the Original Texts each have their unique strengths, the model-generated texts excel in terms of linguistic elegance, offering a clear advantage in text refinement tasks. These results provide a quantifiable metric for evaluating the effectiveness of text refinement models and offer a foundation for more objective and consistent assessments in future research.

## Threats to validity

One potential threat to validity lies in the composition of the datasets used in this study. While our primary datasets—data-ebook and data-UN6—cover a broad range of formal written English, they may still lack sufficient representation of colloquial or domain-specific content such as scientific writing or social media language. Although a portion of the data-ebook set (approximately 10%) includes academic texts sourced from ArXiv, this subset may not be large or diverse enough to fully capture the stylistic and structural characteristics of technical domains. This limitation could affect the generalizability of our findings to highly specialized applications.

Another limitation concerns the range of models used in the evaluation. Our experiments focus on three widely recognized large-scale text generation models: T5, BART, and GPT-3. While these models are strong baselines and remain competitive, the field has seen rapid progress with the emergence of more advanced architectures (e.g., GPT-4, PaLM 2, and Claude). These newer models may demonstrate superior performance on text refinement tasks, and their inclusion could yield different conclusions regarding the relative strengths of various pretraining objectives. We leave a broader benchmarking of newer models as an avenue for future research.

Lastly, while our current study provides a comprehensive evaluation of model outputs in terms of linguistic elegance, semantic consistency, and overall quality, it does not yet explore the downstream utility of the constructed dataset for tasks such as summarization, translation, or essay generation. Including transfer tasks in future work would further strengthen the impact of our method by demonstrating its adaptability across a wider range of natural language processing applications.

## Conclusion

Human writing often exhibits a range of styles and levels of sophistication. However, automated text generation systems typically lack the nuanced understanding required to produce refined and elegant prose. This gap underscores the need for robust text refinement systems that can bridge the divide between ordinary and polished text. This paper introduces a novel context-aware text refinement task aimed at rewriting text to make it more elegant while preserving its original meaning. Text refinement is an essential application for intelligent writing assistants but lacks extensive research in existing literature. To advance research in this task, we explore the text refinement task by: (i) formalizing it as a context-aware sequence-to-sequence text generation problem; (ii) proposing a semi-automatic data labeling method to address the difficulty of manual annotation for refinement data, and constructing datasets for training and evaluating refinement models using this method; (iii) introducing pretraining objectives tailored for the text refinement task and training a series of models on a large-scale English corpus using these objectives as baseline models for the refinement task. Extensive text refinement experiments were conducted based on these baseline models, and the results indicate that fine-tuning the models with the paraphrase objective leads to superior text refinement performance.

Leveraging both human expertise and machine learning techniques presents a promising avenue for achieving this goal. By harnessing human-machine collaboration, we can construct high-quality datasets and develop models that excel in the text refinement task. For future research, investigating text refinement tasks could progress in two main directions. One avenue involves designing automated evaluation metrics suited for text refinement tasks, distinguishing between elegance of expression and semantic consistency. Another direction is to explore aspects of text refinement beyond word usage, such as employing appropriate rhetorical devices to make texts more vivid, and exploring model performance in different languages, including the Chinese context.

## Data Availability

The data that support the findings of this study are available upon request from the corresponding author. Due to privacy and ethical considerations, the data cannot be made publicly available before the project’s completion. Additionally, as we are currently conducting ongoing expansion experiments, some data are not yet ready for public release. However, we are willing to provide selected data upon request by reviewers or editors as necessary.
